# *InteractiveXRDFit*: a new tool to simulate and fit X-ray diffractograms of oxide thin films and heterostructures

**DOI:** 10.1107/S1600576718012840

**Published:** 2018-10-18

**Authors:** Céline Lichtensteiger

**Affiliations:** a DQMP, University of Geneva, 24 Quai Ernest Ansermet, Geneva 4, 1211, Switzerland

**Keywords:** simulation, X-ray diffractograms, heterostructures, epitaxial structures, *c*-axis parameter

## Abstract

*InteractiveXRDFit* is a custom-made MATLAB program that calculates the X-ray diffracted intensity for oxide thin films and heterostructures based on a library of inorganic materials.

## Abstract 2

*InteractiveXRDFit* is a custom-made MATLAB program that calculates the X-ray diffracted intensity for heterostructures.

## 1. Introduction   

With recent advances in deposition techniques, artificial materials can be produced with atomic scale control, creating new functional materials with tailored electronic properties. These fascinating materials can be studied with a plethora of advanced techniques. Independent of their properties, their characterization usually starts with conventional X-ray diffraction (XRD) to attest their crystalline quality. Indeed, most crystal-growth laboratories are equipped with X-ray diffractometers, standard instruments that are relatively inexpensive, fast and non-invasive.

Based on these observations, the program *InteractiveXRDFit* was developed to allow the user to simulate the (00*L*) diffractogram of heterostructures combining different materials grown on different substrates (*L* is defined in Fig. 2). The user can choose the substrate and the different materials composing a heterostructure from a long list of compounds (mainly perovskite oxides), choose between (001) or (111) substrate orientation, and play with the different structural parameters (unit-cell size and number of layers). It is possible to build a superlattice composed of up to three different materials, and to add a top and/or bottom layer (to simulate electrodes, spacers or capping layers). The simulation is quick and allows the user to compare it directly with experimental measurements, so as to give a rapid determination of the crystalline parameters of the sample.

This paper describes the program, how to use it and how it is constructed, with an example for illustration.

## 2. Technical details   

*InteractiveXRDFit* was written using MATLAB R2015b (The MathWorks Inc., Natick, MA, USA) for Mac. It is compatible with Linux, Unix, Mac OS and Windows. The version available at the date of publication of this paper can be found in the supporting information. However, this program is constantly being adapted to suit users’ needs, and anyone interested in using the latest release can obtain it from GitHub. If the user does not already have an account, he/she should start by creating a personal GitHub account (for free): https://github.com. The user should then request access to *InteractiveXRDFit*.

The user can then download every file for the latest version of the program, open MATLAB on his/her computer and run InteractiveXRDFit.m.

## 3. Program use   

**Figure 1:**
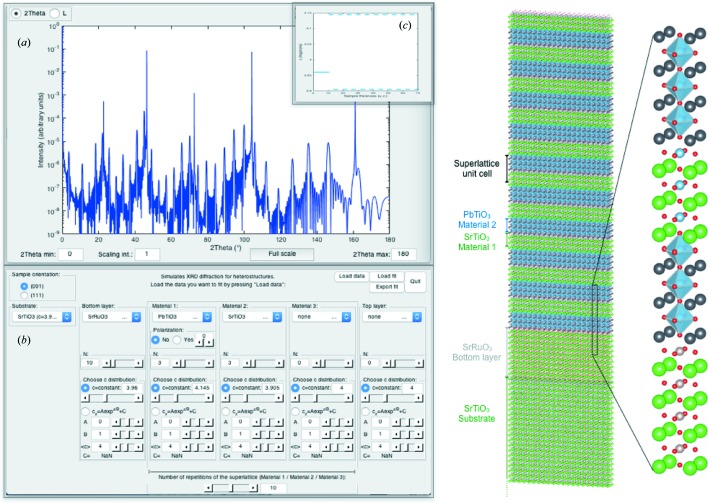
The different program panels. (*a*) The XRD fitting parameters window, (*b*) the fit display and (*c*) the *c* display. The calculated structure is represented on the right-hand side, drawn using *VESTA* (Momma & Izumi, 2011 ▸). It is composed of an SrTiO_3_ substrate (*N* = 2 × 10^4^ unit cells, *c* = 3.905 Å), an SrRuO_3_ bottom layer (*N* = 10 unit cells, *c* = 3.96 Å), and a superlattice comprising 3 unit cells of PbTiO_3_ (*c* = 4.145 Å) and 3 unit cells of SrTiO_3_ (*c* = 3.905 Å) repeated ten times.

Once the user runs InteractiveXRDFit.m, three windows will appear:

(i) the XRD fitting parameters window (Fig. 1 ▸ *a*),

(ii) the *c* display (Fig. 1 ▸ *c*), and

(iii) the fit display (Fig. 1 ▸ *b*).

### 3.1. The XRD fitting parameters window   

From the XRD fitting parameters panel (Fig. 1 ▸ *a*), the user has the following options:

(i) Quit the program.

(ii) Load data to be fitted (has to be a .csv file). When loading data for comparison, two vertical green lines are displayed together with the data (in red) and the simulation (in blue). They delimit the region over which the r.m.s. values are evaluated [see equations (12) and (13)].

(iii) Export the fit (as a .csv file).

(iv) Choose the parameters describing the heterostructure.

The heterostructure can be composed of

(*a*) a substrate,

(*b*) a bottom layer,

(*c*) a superlattice composed of up to three different materials, and

(*d*) a top layer.

Substrates can be chosen from DyScO_3_, GdScO_3_, KTaO_3_, LaAlO_3_, LaSrAlO_4_, LSAT [(LaAlO_3_)_0.3_(Sr_2_AlTaO_6_)_0.7_], NdAlO_3_, NdGaO_3_, Si, SrTiO_3_, TbScO_3_ and YAlO_3_.

Layer materials can be chosen from AlO_2_, BaO, BaTiO_3_, BiFeO_3_, CaCuO_2_, LaAlO_3_, La_2_CuO_4_, LaFeO_3_, LaMnO_3_, LaNiO_3_, La_2_NiMnO_6_, LaO, LSMO (La_0.67_Sr_0.33_MnO_3_), MnO, MnO_2_, MnTiO_3_, NiO_2_, NdNiO_3_, Nd_2_NiMnO_6_, NdO, PbO, PbTiO_3_, (Pb_*x*_Sr_1−*x*_)TiO_3_, Pb(Zr_*x*_Ti_1−*x*_)O_3_, PrBa_2_Cu_3_O_7_, RuO_2_, SmNiO_3_, SrO, SrO_2_, SrRuO_3_, SrTiO_3_, SrVO_3_, TiO_2_, VO_2_, YBa_2_Cu_3_O_7_ and ZrO_2_.

For each layer, the user can define the number of unit cells and the *c*-axis value. The *c*-axis value either can be a constant (in most cases) or can vary as a function of depth within a layer (as will be discussed in the example in §7).

### 3.2. The *c* display   

The *c*-axis value is then displayed on a separate graph (Fig. 1 ▸ *c*) to help visualize the evolution of the *c* axis throughout the heterostructure.

### 3.3. The fit display   

**Figure 2:**
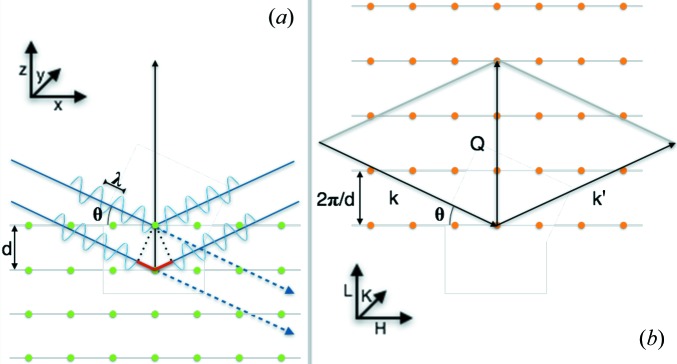
Representation of Bragg’s law in a standard XRD setup in (*a*) direct space and (*b*) reciprocal space.

The XRD intensity is calculated and displayed in the fit display window (Fig. 1 ▸ *b*) after each modification of a parameter, to follow rapidly any changes in intensity as a function of the different parameters and compare the simulations with the measurement. In this window, one can display the data as a function of either 2θ (as defined in Fig. 2 ▸) or reciprocal *L*, change the limits for the graph, and change the scaling of the calculated intensity for better comparison with the measurement.

## 4. Calculations   

### 4.1. XRD measurement geometry   

The calculations use Bragg’s law of reflection, represented schematically in Fig. 2 ▸. A parallel incoming beam is reflected by the atomic planes. Two such beams are represented in blue in Fig. 2 ▸(*a*), the lower one having to cover a longer distance (in red) equal to 2*d*sinθ. The beams are characterized by their wavelength λ and their wavevectors **k** = 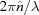. If the path difference corresponds to a multiple of the X-ray wavelength λ, the different beams will interfere constructively, resulting in a peak in the X-ray reflection. This is the famous Bragg diffraction law,
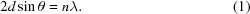
In reciprocal space, this condition is equivalent to **k**′ − **k** = **Q**, where **Q** is a Bravais vector of the reciprocal lattice, as shown in Fig. 2 ▸(*b*). Further details can be found in the work of Ashcroft & Mermin (1976 ▸).

Bragg’s law tells us whether or not the conditions are met for diffraction to be observed. The intensity of this diffraction is calculated below using a bottom-up approach, starting with the contribution from individual atoms, then looking at the contribution from a group of atoms organized into a unit cell, and finally building the whole heterostructure to get the total response of the whole sample.

### 4.2. Atomic scattering factors   

The atomic scattering factors *f* are calculated using the formula 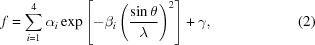
where the values for the parameters α_*i*_, β_*i*_ and γ are found in *International Tables for Crystallography*, Vol. C (Brown *et al.*, 2006 ▸). (Note that the values used are for neutral atoms. The scattering factor values change if one uses ions, but this change will be small and will only affect the relative intensities and not the peak positions.) θ is the diffraction angle and λ the wavelength of the diffracted beam (here λ = 0.15406 nm), as described in Fig. 2 ▸. This is done for a number of atoms: Al, Ba, Bi, Ca, Cu, Dy, Fe, Ga, Gd, K, La, Mn, Nd, Ni, O, Pb, Pr, Ru, Sc, Si, Sm, Sr, Ta, Tb, Ti, V, Y and Zr. Others will be added on request. In the case of alloying on an atomic site, an effective atomic scattering factor is created by taking the weighted average of the atomic scattering factors for the respective individual atoms.

### 4.3. Structure factors   

The structure factor is then obtained for each unit cell using the Wyckoff positions within this cell: 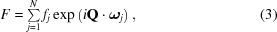
where *N* is the number of atoms in the unit cell and **Q** is a Bravais vector of the reciprocal lattice, also known as the momentum transfer [||**Q**|| = 4πsin(θ)/λ]. Since this program evaluates the Bragg diffraction along the **Q** direction as shown in Fig. 2 ▸(*b*), only the *z* value for the different atoms and the distance *d* between equivalent planes of the corresponding material are needed: 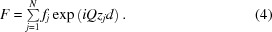
A few examples are described below.

#### 4.3.1. Perovskite structure   

**Figure 3:**
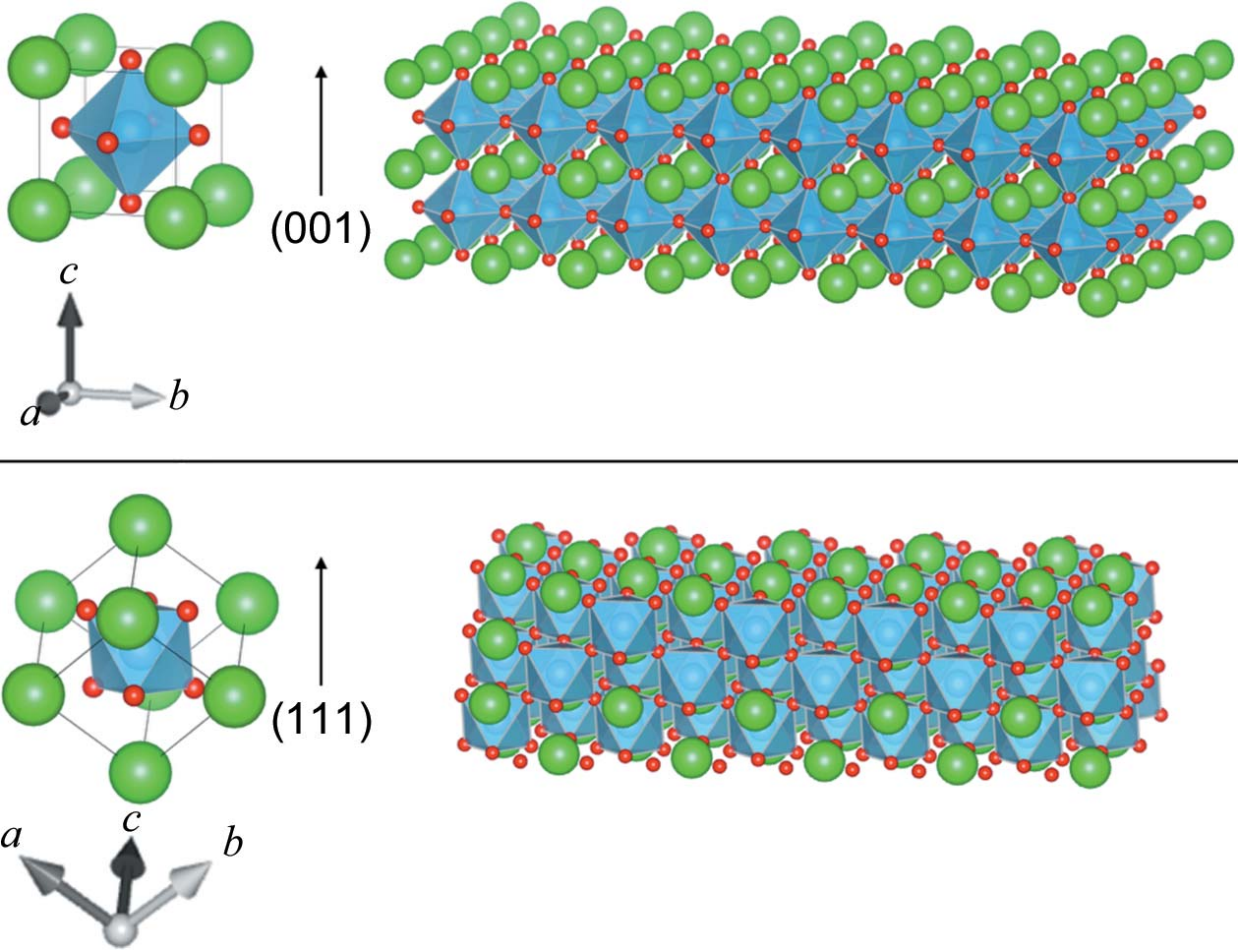
The cubic perovskite structure, (top) in the (001) orientation and (bottom) in the (111) orientation. Drawn using *VESTA* (Momma & Izumi, 2011 ▸).

Most of the materials used in this program have a perovskite structure *AB*O_3_, as shown in Fig. 3 ▸. For simplification, we will consider a simple cubic perovskite structure with unit cell *c*. In the (001) orientation (Fig. 3 ▸, top), the atoms are in the positions shown in Table 1 ▸ (in lattice parameter units *c*).

The structure factor *F* is then 
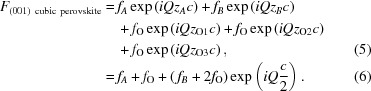



In the (111) orientation (Fig. 3 ▸, bottom), one has to consider not one but three unit cells with the atoms in the positions shown in Table 2 ▸ (in lattice parameter units). In the *x*, *y* and *z* directions, the lattice parameter units are equal to *c*(2)^1/2^, *c*(2)^1/2^ and *c*/(3)^1/2^, respectively, where *c* is the lattice parameter of the unit cell.

One can easily see that the positions along *z* of the groups of atoms (A2 O4 O5 O6 B2) and (A3 O7 O8 O9 B3) are equivalent to (A1O1 O2 O3 B1). We therefore use the first set of five atoms as our unit cell to calculate the structure factor *F*:
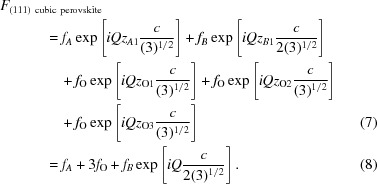



#### 4.3.2. LaSrAlO_4_ substrate   

**Figure 4:**
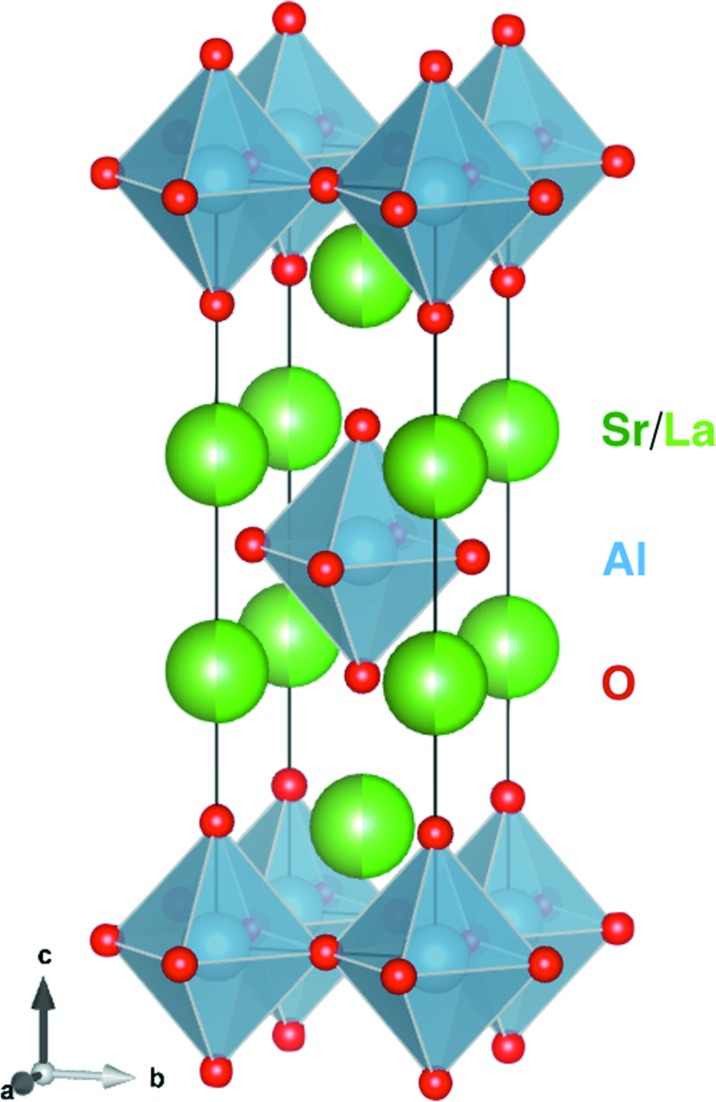
LaSrAlO_4_ is a tetragonal material used as a substrate with *a* = *b* = 3.75576 Å and *c* = 12.6377 Å. Drawn using *VESTA* (Momma & Izumi, 2011 ▸).

LaSrAlO_4_ is a tetragonal mater­ial used as a substrate, with *a* = *b* = 3.75576 Å and *c* = 12.6377 Å, as shown in Fig. 4 ▸. In the (001) orientation, the atoms are at the positions shown in Table 3 ▸ (in lattice parameter units).

The structure factor *F* is then 
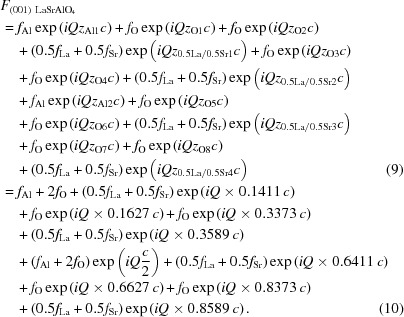



### 4.4. Scattering amplitude and scattered intensity   

In the kinematic theory of diffraction, the scattering amplitude *g* for the whole heterostructure is then obtained by adding the contributions from each layer. However, this neglects the effects associated with the absorption of the beam as it passes through the sample, or the reduction in intensity due to scattering and diffraction. The dynamic theory of diffraction takes these effects into account, but involves a degree of complexity that is beyond the scope of this program (Zachariasen, 1945 ▸). Here, we chose an intermediate approach consisting of modifying the kinematic approach by adding an attenuation factor to take absorption effects into account. The scattering amplitude is then
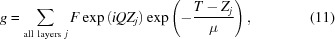
where *Z*_*j*_ is the position of the *j*th layer in the *z* direction, *T* is the total sample thickness and μ is the penetration depth. The first exponential term takes into account the correct phase depending on the position of the considered layer in the structure. The second exponential term takes into account the penetration depth reducing the intensity of the contribution of the deeper layers. A penetration factor μ = 1.5 × 10^4^ Å is used, as it was found to give a better representation for the substrate intensities.

The scattered intensity is then just *I* = gg*, and it is renorm­alized to a maximum intensity equal to 1, allowing comparison with the measurements that are also renormalized.

## 5. Program organization   

An important part of the program is dedicated to the graphical user interface (GUI) providing point-and-click control of the program, so that the user can run the application with no need to learn the MATLAB language or type commands.

Other parts of the program calculate the atomic scattering factors, structure factors, scattering amplitude and scattered intensity of the heterostructure built by the user. In order to save time, the substrate scattering amplitudes have all been calculated beforehand and are uploaded by the program.

The program is composed of the following MATLAB scripts and functions:

(i) InteractiveXRDFit.m. This is the heart of the program. It initializes all the variables and creates the GUI.

(ii) Substrate.m. This script loads the correct substrate data and intensity (from Substrates.mat) depending on the user’s choice.

(iii) GenerateSubstrates.m. This script generates the XRD intensities for different substrates. It is never called from the other scripts. This is the script that was used to calculate the substrate scattering amplitudes beforehand in order to save time (saved in Substrates.mat). It is made available only for the sake of completeness.

(iv) PlotFitAndData.m. This script plots the calculated XRD intensities on the fit display panel. If the user has loaded a measurement for comparison, this script also displays the measured XRD intensities, together with the lines delimiting the region over which the RMS (root mean square) is evaluated. On the *c* display panel, it plots the *x*-axis profile of the simulated heterostructure.

(v) ProgcNSimu.m. This script calculates the XRD intensity and *c*-axis profile of the heterostructure defined by the user. It is used every time the user modifies a parameter in the XRD fitting parameters panel.

(vi) expzm.m. This function calculates the contribution from each layer material to the diffracted intensity.

(vii) FLayer.m. This function calculates the form factors of the different materials depending on their crystallographic structures. ‘*z*’ are the out-of-place atomic positions in the different unit cells, expressed in relative unit cells.

(viii) StructureFactor.m. This function calculates the structure factor for materials with a perovskite structure (other structures are calculated directly within FLayer.m).

(ix) AtomicScatteringFactor.m. This function calculates the different atomic scattering factors used in this program, based on *International Tables for Crystallography*, Vol. C.

(x) RMS.m. This script is used to calculate the RMS of the pairwise differences of the fit and measurement, 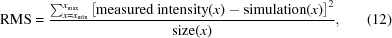
and RMS(log), the RMS of the pairwise differences of the log of the fit and the log of the measurement, 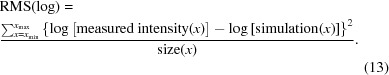
The user can change the region delimited by *x*_min_ and *x*_max_ over which the RMS and RMS(log) values are calculated (shown by vertical green lines on the fit display panel). This is done only if the user loads measured data, to compare them with the fit.

## 6. Program limitations and future developments   

As discussed in §4.4, we chose an intermediate approach between the two limits of the kinematic and dynamic X-ray diffraction theories. Additionally, there are many factors that affect the diffracted intensity which are not taken into account here, such as beam size, sample alignment, surface and interface roughness, relaxation and rumpling, mosaicity, off-stoichiometry, vacancies, vicinality, and defects. Some of these factors will be added to future versions of the program. These simplifications were implemented in order to obtain a practical program that reproduces the main features of an XRD pattern, allowing the user to simulate quickly complex heterostructures with a limited number of adjustable parameters describing in the most convenient way the structure of the desired materials.

## 7. Example: constant *versus* depth-varying *c*-axis value   

**Figure 5:**
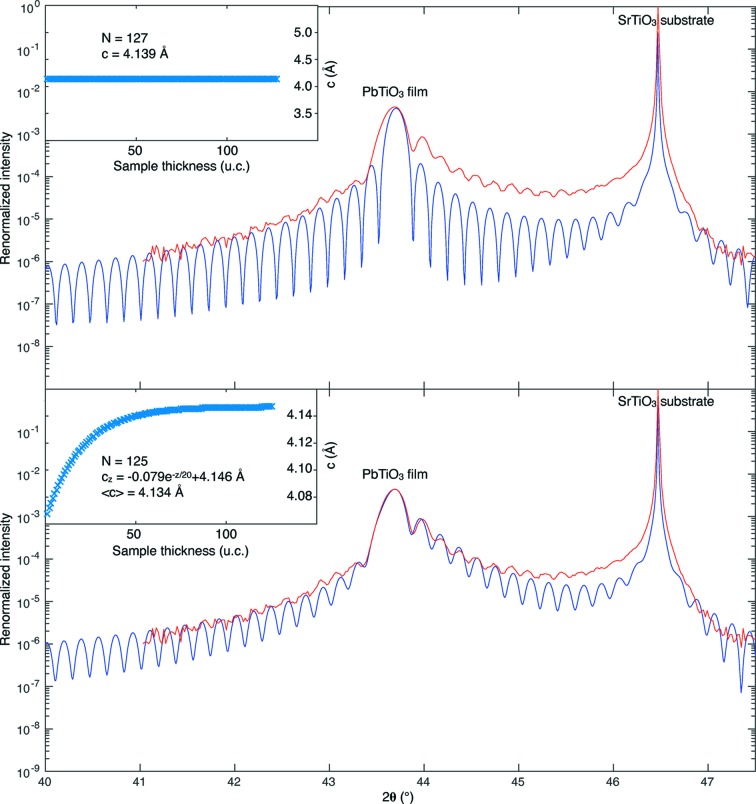
Comparison between two fits for a PbTiO_3_ thin film on a (001)-oriented SrTiO_3_ substrate. The measurement is in red and the simulation in blue. The simulation in the top part is with a constant lattice parameter, while the bottom part corresponds to a depth-varying *c*-axis value described with an exponential function. The respective film lattice parameters are shown as insets. This clearly demonstrates a better agreement between the measurement and the simulation when the depth-varying *c*-axis value is chosen. From Weymann *et al.* (2018 ▸).

An interesting example is shown in Fig. 5 ▸. In a series of PbTiO_3_ samples grown on SrTiO_3_ substrates, standard simulations (*i.e.* with a constant *c*-axis value) were not giving satisfactory results. This is shown in Fig. 5 ▸ (top), where a simulation for PbTiO_3_ with *N* = 127 unit cells and *c* = 4.139 Å (in blue) is compared with the measurement (in red). The value of *c* is determined by the PbTiO_3_ peak position and the number of unit cells is optimized to fit the finite size oscillations. However, the width of the simulated film peak is narrower than the measured one, and the oscillations to the right of the film peak are less intense than the measured ones.

The option to fit the diffractogram using a depth-varying *c*-axis value described with an exponential function was added to the program. The result obtained for *N* = 125 and *c*_*z*_ = −0.079exp(−*z*/20) + 4.146 Å (corresponding to an average value of *c* = 4.134 Å) is shown in Fig. 5 ▸ (bottom), demonstrating the better agreement between the simulation and the measurement. In addition to the good match with the finite size oscillations and the peak position, the shape of the peak and the intensity of the oscillations on the right of the film peak are also now perfectly matching.

## 8. Conclusions   

The program *InteractiveXRDFit* was developed to answer the need for users to simulate rapidly the (00*L*) diffractogram of heterostructures combining different materials grown on different substrates.

Other fitting programs are already available and they are efficient, but they may lack some flexibility with some of the parameters that this program allows to be modified. For example, *Epitaxy* (https://www.malvernpanalytical.com/en/products/category/software/X-ray-diffraction-software/epitaxy) from Malvern Panalytical allows thorough analysis of X-ray diffraction data with efficient fitting procedures, taking the instrumental details (background, divergence and intensity) into account and allowing the user to determine the sample structure properties (layer density, thickness and roughness), but the materials data remain fixed as they are in the materials database. *SUPREX*  (Fullerton *et al.*, 1992 ▸) and *CADEM*  (Komar & Jakob, 2017 ▸) are other examples of programs calculating X-ray diffraction from epitaxial layers, with *SUPREX* very useful for high-*T*_c_ superconductor superlattices, and *CADEM* focusing on half-Heusler superlattices such as HfNiSn/TiNiSn. While *SUPREX* was written in Fortran and in Turbo Pascal, *CADEM* was written using MATLAB and can be adapted to other materials as well. The interested user might also want to try *COBRA* (Yacoby *et al.*, 2002 ▸), a phase-retrieval algorithm that has proved to be useful in the study of buried interfaces in epitaxial heterostructures; *GenX* (http://genx.sourceforge.net), a versatile program using the differential evolution algorithm for fitting X-ray and neutron reflectivity data as well as surface X-ray diffraction data (Björck & Andersson, 2007 ▸); or the *xrayutilities* (https://xrayutilities.sourceforge.io/index.html) Python scripts for assisting X-ray diffraction experiments.

*InteractiveXRDFit* is simple to use and has the advantage of allowing the user to carry out rapid simulations of different heterostructures and see how changing the parameters modifies the diffractogram for a better understanding of the measurements.

Additionally, it can be easily adapted to the user’s needs, with more materials and functionalities added on demand. Requests for any new requirements are most welcome.

## Supplementary Material

App allowing direct installation of the free-standing executable of the program. DOI: 10.1107/S1600576718012840/vh5084sup1.zip

MATLAB main program InteractiveXRDFit.m, 9 MATLAB sub-programs and file containing the calculated intensities of the different substrates (Substrates.mat). DOI: 10.1107/S1600576718012840/vh5084sup2.zip

## Figures and Tables

**Table 1:** Relative positions (in lattice parameter units) of the atoms in the (001)-oriented cubic perovskite structure

	*x*	*y*	*z*
*A*	0	0	0
*B*	1/2	1/2	1/2
O1	1/2	1/2	0
O2	0	1/2	1/2
O3	1/2	0	1/2

**Table 2:** Relative positions (in lattice parameter units) of the atoms in the (111)-oriented cubic perovskite structure

	*x*	*y*	*z*
*A*1	0	0	0
O1	1/2	0	0
O2	1/4	(3)^1/2^/4	0
O3	−1/4	(3)^1/2^/4	0
*B*1	1/2	(3)^1/2^/6	1/2
			
*A*2	0	(3)^1/2^/3	1
O4	1/4	(3)^1/2^/12	1
O5	3/4	(3)^1/2^/12	1
O6	1/2	(3)^1/2^/3	1
*B*2	0	0	3/2
			
*A*3	1/2	(3)^1/2^/6	2
O7	1/4	5(3)^1/2^/12	2
O8	−1/4	5(3)^1/2^/12	2
O9	0	(3)^1/2^/6	2
*B*3	0	(3)^1/2^/3	5/2

**Table 3:** Relative positions (in lattice parameter units) of the atoms in the (001)-oriented LaSrAlO_4_ substrate

	*x*	*y*	*z*
Al1	0	0	0
O1	0	1/2	0
O2	1/2	0	0
0.5La/0.5Sr1	1/2	1/2	0.1411
O3	0	0	0.1627
O4	1/2	1/2	0.3373
0.5La/0.5Sr2	0	0	0.3589
Al2	1/2	1/2	1/2
O5	0	1/2	1/2
O6	1/2	0	1/2
0.5La/0.5Sr3	0	0	0.6411
O7	1/2	1/2	0.6627
O8	0	0	0.8373
0.5La/0.5Sr4	1/2	1/2	0.8589
