# Supplementary figures and images for: InteractiveXRDFit: a new tool to simulate and fit X-ray diffractograms of oxide thin films and heterostructures
Source: J Appl Crystallogr. 2018 Oct 18;51(Pt 6):1745–51. doi: 10.1107/S1600576718012840 (PMC6276276; doi:10.1107/S1600576718012840)

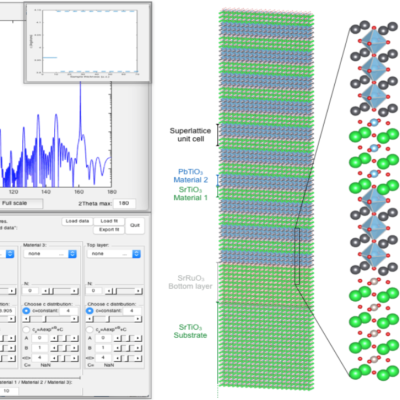

Supplement: Supplementary file 1 [file j-51-01745-sup1.zip › MyAppInstaller_web.app/Contents/Resources/splash.png]
